# Inappropriate Multi-Target Stool DNA Use for Colorectal Cancer Screening: Risks, Compliance, and Outcomes

**DOI:** 10.7759/cureus.40506

**Published:** 2023-06-16

**Authors:** Nicholas J Lazar, Ali Khreisat, Roa'a AlKloub, Alsadiq Al-Hillan, Michael Duffy

**Affiliations:** 1 Internal Medicine, Beaumont Hospital, Royal Oak, USA; 2 Gastroenterology and Hepatology, Beaumont Hospital, Royal Oak, USA

**Keywords:** cologuard®, off-label mt-sdna, michigan, colon cancer screening, stool-based cancer screening, multi-target stool dna, colorectal cancer screening, colon cancer prevention

## Abstract

Background

Inappropriate or "off-label" use of multi-target stool DNA (mt-sDNA) tests refers to their use in patients for whom colonoscopy or no testing at all is warranted. Examples include a positive family history of colorectal cancer, a history of inflammatory bowel disease, or medical issues necessitating diagnostic colonoscopy, among others. Current understanding of off-label mt-sDNA use for colorectal cancer screening, its associated risks, and outcomes is lacking. We examined off-label mt-sDNA prescription and compliance with testing in an outpatient setting in southeast Michigan.

Aims

The primary aims of the study were determining the extent of off-label mt-sDNA testing and compliance, and results of all testing, as well as demographic factors associated with off-label prescriptions. The secondary aims were to examine explanations for incomplete testing and factors contributing to successful completion.

Methods

Using a retrospective design, we identified mt-sDNA orders from outpatient internal medicine clinics between January 1, 2018, to July 31, 2019, to evaluate the proportion of off-label mt-sDNA, results of testing, and follow-up colonoscopies up to one year after order placement. Patients were categorized as “off-label” if any inappropriate criteria were met. Statistical analysis was performed for primary and secondary outcomes.

Results

From 679 mt-sDNA orders within the study period, 81 (12.1%) had at least one off-label criterion for testing. In total, 404/679 (59.5%) patients completed testing. Lack of follow-up comprised the majority of incompletions (216/275; 78.6%). Only 52 (70.3%) out of 74 positive results were followed by diagnostic colonoscopy. Retired employment status (OR = 1.87; 95%CI, 1.17-2.98; P = 0.008) and age of 76 years or older (OR = 2.28; 95%CI, 0.99-5.21; P = 0.044) were significantly associated with increased risk of off-label mt-sDNA prescription. Increasing age range was associated with higher test completion (χ2 (5) = 12.085, p = 0.034). Multinomial logistic regression revealed an increasing age range (OR = 1.29; 95% CI, 1.09-1.54; P = 0.004), predictive of a positive mt-sDNA result for both groups. There was no significant difference between off-label or on-label groups in the mean number of resected polyps or pathology scores on follow-up colonoscopy.

Conclusions

Off-label mt-sDNA use remains a concern in the outpatient setting. Compliance for test completion and follow-up colonoscopy for positive results require further improvement. Our findings shed new light on the factors associated with off-label testing while reiterating its burden. We also describe common reasons for incomplete tests in an attempt to augment future colorectal cancer (CRC) screening initiatives.

## Introduction

Colorectal cancer (CRC) screening plays a crucial role in early detection and prevention of one of the most common cancers worldwide [1, 2]. Cologuard® (Exact Sciences Corp., Madison, Wisconsin, United States), a widely used multi-target stool DNA (mt-sDNA) test for CRC screening, has gained popularity due to its convenience and non-invasive nature [3]. However, despite its advantages, Cologuard has some limitations that hinder its effectiveness as a gold-standard screening method [4,5]. This research paper aims to investigate the burden of off-label mt-sDNA testing, identify factors contributing to inappropriate orders, examine their associations with patient outcomes, and explore reasons for test incompletion to identify possible barriers to adherence and guide future interventions.

Cologuard has demonstrated high sensitivity in detecting CRC and advanced adenomas, providing valuable opportunities for early detection [4,6,7]. Nevertheless, it is plagued by a high false-positive rate and low positive predictive value (PPV), occasionally resulting in unnecessary follow-up interventions [4,8]. Unlike the gold-standard colonoscopy, mt-sDNA lacks the ability to intervene and remove identified abnormalities during the screening process. Consequently, a positive mt-sDNA result necessitates further diagnostic evaluation through colonoscopy, potentially leading to delayed initiation of appropriate therapy.

Compliance with stool-based testing remains a common concern. While approximately 80% of individuals complete their orders, a significant percentage of positive results do not receive subsequent follow-up colonoscopies [9-11]. This failure to complete the necessary diagnostic procedure can be attributed to various factors, including patient-related barriers and healthcare system inefficiencies [9,12-14]. Importantly, delaying follow-up colonoscopy for positive tests may result in an increased risk of advanced findings or CRC [15,16].

In recent years, studies have explored different strategies to improve compliance with mt-sDNA testing. Outreach initiatives, such as virtual chart reminders, office calls, and office-based education (referred to as "inreach"), have shown promising results in enhancing adherence [17-20]. However, there is limited research investigating the reasons for incomplete testing and other factors predicting completion, underscoring the need for further investigation.

Furthermore, recent studies have revealed a substantial burden of off-label prescribing of mt-sDNA [21-23]. Off-label use refers to the application of a medical test or treatment for indications not specifically approved by regulatory authorities. Patients who receive off-label Cologuard testing, either due to age restrictions or other factors, may face an increased risk of positive test results [19]. Consequently, the appropriate initiation of therapy may be delayed, highlighting the significance of understanding the factors associated with off-label testing.

This study aims to expand the knowledge regarding factors associated with off-label mt-sDNA testing. By exploring variables such as race, employment status, marital status, median income, and other demographic factors, the study seeks to identify potential predictors of off-label orders and the risk of positive test results for these cases. Additionally, the study aims to determine reasons for test incompletion, providing insights into possible barriers to adherence, and guiding future interventions to improve compliance with CRC screening. By addressing these outcomes, we can enhance the effectiveness of mt-sDNA as a non-invasive screening tool and ensure the timely initiation of appropriate therapy for patients at risk of CRC.

## Materials and methods

This study was conducted in Corewell Health East William Beaumont University Hospital, Royal Oak, Michigan, United States. Seven outpatient internal medicine clinics including Southfield, Royal Oak, Taylor, Canton, Detroit, Bloomfield Hills, and Hazel Park (comprising over 60 primary care physicians) affiliated with a large southeast Michigan healthcare system, Corewell Health, were randomly selected and included in the study. We queried Epic® electronic medical record (EMR) (Epic Systems Corporation, Verona, Wisconsin, United States) for mt-sDNA orders placed during visits at these facilities between January 1, 2018, to July 31, 2019, yielding 679 patients. This was the final study population. All follow-up data were collected up to one year after mt-sDNA result. The study was approved by the Institutional Review Board, Corewell Health East William Beaumont Hospital Research Institute (approval number: 2022-018 dated February 21, 2022).

A manual chart review of deidentified records was done by two separate study authors (AK, NL) to gather data that could not be readily queried by Epic warehouse search. This included data such as narrative endoscopy reports, mt-sDNA results scanned into the EMR under “Media,” etc. Baseline characteristics included sex, age of patient at the time of the mt-sDNA order, race, ethnicity, employment status, marital status, median household income, and tobacco use. Age was stratified into cohorts and average household income was determined by ZIP (Zone Improvement Plan) code according to the United States Census Bureau then stratified into income ranges.

Patients were divided into “on-label” or “off-label” groups respectively by absence or presence of any off-label criteria. There were six categories of off-label criteria for inappropriate mt-sDNA orders: (1) screening colonoscopy necessary over mt-sDNA for history of high-risk adenoma, personal history of CRC, personal history of inflammatory bowel disease or polyposis syndromes, (2) family history of CRC, (3) diagnostic colonoscopy indicated at time of mt-sDNA order given suspicion for gastrointestinal bleeding, uninvestigated chronic diarrhea, iron deficiency anemia, positive fecal immunochemical test (FIT) or fecal occult blood test (FOBT) within one year prior to mt-sDNA order, (4) negative colonoscopy within 10 years prior to mt-sDNA order, (5) negative FIT or FOBT within one year prior to mt-sDNA order, and (6) age less than 45 years or greater than 85 years precluding screening.

International Classification of Diseases, 10th Revision (ICD-10) codes were queried for diagnoses linked to the mt-sDNA order, diagnoses present on outpatient visit problem lists, past medical history, and family history for each patient. We gathered results of FOBTs one year prior to the mt-sDNA order, previous mt-sDNA results three years prior to the study period order, and colonoscopy results up to 10 years prior to the study period.

Results of mt-sDNA were categorized as “negative”, “positive” or “indeterminate without resubmission.” If the mt-sDNA order was not completed, the documented reason was collected and categorized as follows; (1) the patient was lost to follow-up and/or the mt-sDNA order expired, (2) the patient later refused testing after the initial order and the order was subsequently canceled, (3) insurance-related issues, or (4) the order was erroneously entered by the provider.

Results of follow-up colonoscopies for positive mt-sDNA samples were collected for all patients. We used endoscopy reports and pathology reports to gather the number of polyps resected and the histology grade for each polyp removed. For polyp histology, we scored results for degree of dysplasia on pathology reports as follows: (1) One point for hyperplastic histology, (2) two points for tubular histology, (3) three points for sessile serrated adenoma, (4) four points for tubulovillous histology, and (5) five points for high-grade dysplasia.

Primary outcomes were percentage of all mt-sDNA tests meeting one off-label criterion or more, risk factors of off-label testing, percent completion of all mt-sDNA orders and outcomes of testing, explanation for incomplete tests, predictors of a positive test, and polyp number and histology score on follow-up colonoscopies for both groups. Secondary outcomes were associations between completion of mt-sDNA order and baseline characteristics (age range, sex, race, ethnicity, employment status, marital status, median income range) as well as associations of smoking status with the number of resected polyps and pathology score. Statistical analyses were performed using IBM SPSS Statistics for Windows, Version 29.0 (Released 2022; IBM Corp., Armonk, New York, United States).

## Results

Demographic information

A total of 679 patients had mt-sDNA orders within the study time period. Demographic information for both groups including sex, median age at mt-sDNA order, percentage of patients within each cohort, race, ethnicity, marital status, employment status, tobacco use, and percentage of patients within each median annual household income range are shown in Table 1. Sex proportion was similar between groups, each with a majority of female patients.

**Table 1:** Demographic Information for Off-Label and On-Label mt-sDNA Patients mt-sDNA: multitarget stool DNA

Category		Off-Label, n (%)	On-Label, n (%)
Gender	Female	53 (64.6%)	391 (65.5%)
	Male	29 (35.4%)	206 (34.5%)
Median age in years (SD)		65 (8.2)	62 (7.8)
Age range (Years)	≤55	13 (15.9%)	151 (25.3%)
	56-60	9 (11.0%)	105 (17.6%)
	61-65	22 (26.8%)	122 (20.4%)
	66-70	19 (23.2%)	114 (19.1%)
	71-75	11 (13.4%)	78 (13.1%)
	≥76	8 (9.8%)	27 (4.5%)
Race	Asian	0 (0.0%)	13 (2.2%)
	Black or African American	13 (15.9%)	100 (16.8%)
	Other	1 (1.2%)	19 (3.2%)
	Unavailable	1 (1.2%)	1 (0.2%)
	White or Caucasian	67 (81.7%)	464 (77.7%)
Ethnicity	Arab/Middle Eastern Descent	0 (0.0%)	2 (0.3%)
	Hispanic/Latino	1 (1.2%)	8 (1.3%)
	Non-Hispanic/Latino	73 (89.0%)	506 (84.8%)
	Other	7 (8.5%)	70 (11.7%)
	Unavailable	1 (1.2%)	11 (1.8%)
Employment Status	Unemployed or disabled	17 (20.7%)	128 (21.4%)
	Full Time	12 (14.6%)	189 (31.7%)
	Part Time	3 (3.7%)	14 (2.3%)
	Retired	41 (50.0%)	208 (34.8%)
	Self Employed	5 (6.1%)	19 (3.2%)
	Unknown	4 (4.9%)	39 (6.5%)
Marital Status	Divorced/Separated	14 (17.1%)	112 (18.8%)
	Married	36 (43.9%)	294 (49.2%)
	Single	20 (24.4%)	117 (19.6%)
	Unknown	1 (1.2%)	8 (1.3%)
	Widowed	11 (13.4%)	66 (11.1%)
Median Annual Household Income Ranges (US Dollars)	15000 - 24999	0 (0.0%)	6 (1.0%)
	25000 - 34999	4 (4.9%)	43 (7.2%)
	35000 - 49999	3 (3.7%)	24 (4.0%)
	50000 - 74999	54 (65.9%)	380 (63.7%)
	75000 - 99999	17 (20.7%)	112 (18.8%)
	100000 - 149999	4 (4.9%)	27 (4.5%)
	150000 - 199999	0 (0.0%)	5 (0.8%)
Smoking History	Never smoker/Former smoker/Active smoker/Unknown	38 (46.3%), 34 (41.5%), 9 (11.0%), 1 (1.2%)	271 (45.4%), 204 (34.2%), 117 (19.6%), 5 (0.8%)

Primary outcomes

The results indicated 597 (87.9%) patients had an appropriate mt-sDNA order, and 82 (12.1%) had at least one off-label indication for mt-sDNA. Positive family history was the most common off-label indication, comprising 34 (41.5%) off-label patients. Fourteen (17.1%) off-label patients had a negative screening colonoscopy within 10 years of mt-sDNA order. Diagnostic colonoscopy was warranted in 12 (14.6%) patients based on their symptoms and/or order-linked diagnoses. Seven (8.5%) patients would have needed screening colonoscopy based on medical history, and similarly seven (8.5%) patients had a negative mt-sDNA or FOBT prior to the mt-sDNA order in the study period. Only one (1.2%) patient met off-label criteria for advanced age precluding screening. The remaining seven (8.5%) patients had more than one off-label indication (Table 2).

**Table 2:** Off-Label mt-sDNA Indications FIT: fecal immunochemical test; FOBT: fecal occult blood test

Off-Label Indications	n (%)
Positive family history	34 (41.5%)
Screening colonoscopy indicated	7 (8.5%)
Diagnostic colonoscopy indicated	12 (14.6%)
Recent negative colonoscopy	14 (17.1%)
Recent negative FIT/FOBT	7 (8.5%)
Age precludes screening	1 (1.2%)
Patients with >1 off-label indication	7 (8.5%)

The two baseline characteristics with significantly increased risk of off-label ordering were retired employment status (OR = 1.87; 95% CI, 1.17-2.98; P = 0.008) and age range of 76 years or older (OR = 2.28; 95% CI, 0.99-5.21; P = 0.044). There was no difference in risk of off-label order for either gender (P = 0.878).

Of all 679 patients, 404 (59.5%) completed the mt-sDNA order, and 275 (40.5%) did not. Across groups, the majority of incomplete orders were attributed to order expiration and/or lack of follow-up within the study time period (n=216; 78.5%). The remaining explanations in descending order of frequency were patient refusal after the order placement, order cancellation, erroneous order placement, and insurance coverage issues (Table 3).

**Table 3:** Compliance, Results, and Explanations for Incomplete mt-sDNA Testing mt-sDNA: multi-target stool DNA

Outcomes		Off-Label, n (%)	On-Label, n (%)	Total, n (%)
mt-sDNA compliance	Completed	47 (57.3%)	357 (59.8%)	404 (59.5%)
	Incomplete	35 (42.7%)	240 (40.2%)	275 (40.5%)
mt-sDNA result	Negative	37 (78.7%)	285 (79.8%)	322 (79.7%)
	Positive	9 (19.1%)	65 (18.2%)	74 (18.3%)
	Indeterminate without resubmission	1 (2.1%)	7 (2.0%)	8 (2.0%)
Reason for mt-sDNA incompletion	Follow-up/order expired	25 (71.4%)	191 (79.6%)	216 (78.6%)
	Refusal/order canceled	7 (20.0%)	25 (10.4%)	32 (11.6%)
	Insurance issues	0 (0.0%)	10 (4.2%)	10 (3.6%)
	Erroneous order	3 (8.6%)	14 (5.8%)	17 (6.2%)
Underwent follow-up colonoscopy for positive mt-sDNA		7 (77.8%)	45 (69.2%)	52 (70.3%)

In total, 322 (79.7%) mt-sDNA samples were negative and 74 (18.3%) were positive, including one initially indeterminate result that was successfully resubmitted. The remaining eight indeterminate results were not resubmitted. Percentages of positive, negative, and indeterminate sample results were similar between off-label and on-label groups as shown in Table 3.

Upon multinomial logistic regression for baseline characteristics and sample results, only increasing age range (OR = 1.29; 95% CI, 1.09-1.54; P = 0.004) was associated with a higher risk of positive mt-sDNA. No significant association was observed for either gender, any racial category, tobacco use status, or off-label subcategory.

The median time from test order to result was 27 days (IQR 19-49), and the median time from positive test to follow-up colonoscopy was 45 days (IQR 28-86). Fifty-two (70.3%) of the 74 positive mt-sDNA orders were followed by diagnostic colonoscopy within the study time period. An additional 15 patients (four meeting off-label criteria) who did not complete mt-sDNA testing for various reasons incidentally underwent screening colonoscopy within the study time period. The results of all colonoscopy reports are included in Table 4.

**Table 4:** Polyp and Pathology Results

Outcomes	Off-Label	On-Label
Number of resected polyps on colonoscopy (mean)	13 (1.18)	139 (2.48)
Mean pathology score of resected polyps	29.14	32.01
Number of patients with high-risk adenoma	1	11

**Figure 1:**
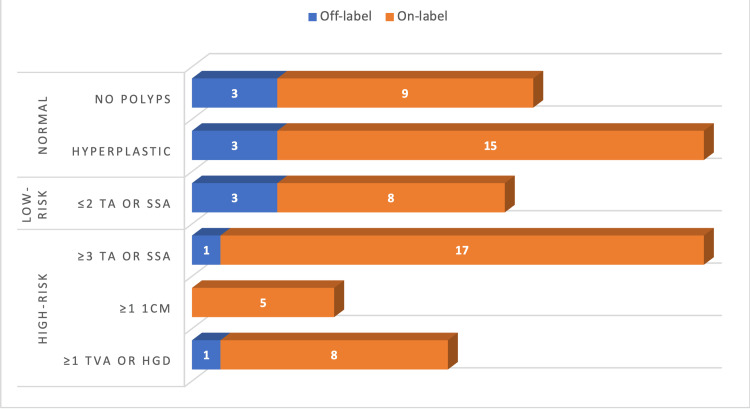
Endoscopy Findings for On-Label and Off-Label Patients “Normal” defined as no polyps or hyperplastic pathology; “Low-risk” defined as two or fewer polyps with tubular adenoma (TA) or sessile serrated adenoma (SSA); “High-risk” defined as either three or more TA or SSA, any polyp greater than one centimeter, or one or more polyp with tubulovillous adenoma (TVA) or high-grade dysplasia (HGD).

Figure 1 shows the frequency of normal, low-risk, and high-risk findings of colonoscopy. Solely considering findings for on-label patients with positive mt-sDNA results who subsequently underwent follow-up colonoscopy, the PPV of mt-sDNA for detecting high-risk adenoma was 46.7% (21/45).

Secondary outcomes

Chi-square tests were conducted to examine the association between demographic data and mt-sDNA completion rates. Age range was significantly associated with test completion (χ2 (5) = 12.085, p = 0.034), with older age ranges completing testing more frequently. However, there was no significant association between test completion with sex (χ2 (1) = 1.030, p = 0.310), race (χ2 (4) = 2.915, p = 0.572), ethnicity (χ2 (5) = 2.514, p = 0.774), employment status (χ2 (5) = 9.048, p = 0.107), marital status (χ2 (4) = 5.299, p = 0.265), or median income range (χ2 (6) = 2.320, p = 0.888).

The average number of resected polyps on follow-up colonoscopy for patients with on-label mt-sDNA was 139 (mean 2.48) and the average for off-label patients was 13 (mean 1.18), implying that the average number of resected polyps for patients with on-label mt-sDNA is greater than average for patients with off-label mt-sDNA. The pathology score average for patients with on-label mt-sDNA was 51 (mean 32.01) and the pathology score average for patients with off-label mt-sDNA was 11 (mean 29.14). This implies that the pathology score average for patients with on-label mt-sDNA is greater than the pathology score average for patients with off-label mt-sDNA (Table 4).

However, an independent sample t-test was conducted to examine the difference in the number of polyps on colonoscopy reports between on-label and off-label mt-sDNA orders. The results revealed that there was no significant (t (65) = 1.950, p = 0.055) difference in the number of polyps on colonoscopy report between on-label (n = 56, mean = 2.48) and off-label (n = 11, mean = 1.18). Also, a Mann-Whitney U test was conducted to examine the difference in the pathology of resected polyps between on-label and off-label mt-sDNA tests. The results established that there was no significant (Z-score = -0.496, p = 0.620) difference in the pathology of resected polyps between on-label (n = 51, mean rank = 32.01) and off-label (n = 11, mean rank = 29.14).

## Discussion

Our study results reaffirm the burden of off-label mt-sDNA testing summarized in the existing literature. Inappropriate use of CRC screening tools (FIT, FOBT, and mt-sDNA) is a well-documented phenomenon, with off-label prescribing for mt-sDNA comprising roughly 15% of orders [8,21]. We discovered a similar pattern with 12.1% of patients meeting at least one off-label criterion, most commonly family history necessitating screening colonoscopy. Among the examined baseline characteristics, age range of 76 years or older (OR 2.28) and retired employment status (OR 1.87) showed a significantly increased risk of off-label prescription. Increasing age range (P = 0.002) was also the only observed predictor of a positive test for both groups. Our results are in keeping with a recent study by Agarwal et al., though employment status was not specifically examined [21].

Unsurprisingly, patients in older age groups are presumably more likely to have cumulative health circumstances warranting diagnostic and/or screening colonoscopy or may have aged out of screening entirely. The potential relationship between retired employment status and risk of off-label mt-sDNA prescription makes rational sense given the average retirement age, though warrants further investigation in future studies. Neither female or male sex nor any of the other examined demographic variables conferred significant risk of off-label prescribing in our study. These findings reinforce the importance of diligence when choosing a means of CRC screening for older patients.

Another well-documented issue is compliance with mt-sDNA testing [9,13,17], including both completion of ordered tests and definitive follow-up colonoscopy in the event of a positive test. Our results showed a 59.5% completion rate of all ordered mt-sDNA tests, a figure slightly lower than reported rates [3,17,21]. The proportions of positive and negative tests did not differ between groups, in contrast with a recent study that estimated a three-fold high risk of positive results for off-label patients [21].

Lack of follow-up and subsequent order expiration was the most common reason for non-completion in our population. Both outreach and “inreach” methods have been discussed in previous studies with improvement in mt-sDNA adherence rates [11,17,19], which argues for a more robust and widespread adoption of such practices. Patient refusal was the second most common reason for the non-completion of tests in our population, likely representing a multifactorial issue for patients and ordering providers. This phenomenon stresses the value of a productive, trusting relationship between patient and physician to address barriers and provide adequate education. Interestingly, increasing age range was the only factor significantly associated with higher test completion from our population.

The median duration for both mt-sDNA order to completion and positive result to follow-up colonoscopy were consistent with previously reported figures [13,17,21]. Of all patients with a positive mt-sDNA, 70.3% underwent follow-up colonoscopy within the study time period. Past studies have reported completion rates for follow-up colonoscopy ranging anywhere from 55-100% [3,13,16,17,21], though heterogeneity in methodologic factors likely accounts for observed differences.

Notably, there was no significant difference in the mean number of resected polyps or mean pathology score from follow-up colonoscopies between on-label and off-label groups. Smoking status also failed to influence these outcomes. The positive predictive value of mt-sDNA to detect high-risk adenoma in our population was 46.7%, echoing previous data and highlighting the importance of using mt-sDNA solely for screening purposes [3-5].

Our study strengths include comprehensive data collection, analysis of baseline characteristics and their relationships with off-label testing and compliance, and the inclusion of endoscopy and pathology results between groups. Older age groups may be at higher risk for inappropriate testing and positive results, thus necessitating follow-up endoscopy regardless. This underscores the need for diligence on the part of healthcare providers when choosing a CRC screening test for older patients in these age groups. Conversely, greater effort should be focused on improving compliance for younger age groups through multimodal outreach and “inreach” programs. While off-label testing has been associated with a higher risk of a positive mt-sDNA result, our results failed to show a significant difference. Additional investigation into employment status and its relationships with off-label mt-sDNA prescribing is warranted.

We acknowledge the limitations of our study, including the retrospective design, and that our patient population is confined to southeast Michigan, possibly affecting the generalizability of our findings. We also recognize that a sizable portion of our data is descriptive and that the coronavirus disease 2019 (COVID-19) pandemic could have played a confounding role with respect to rates of mt-sDNA completion and subsequent follow-up testing.

## Conclusions

Our results contribute to the wealth of information on mt-sDNA compliance and the evolving understanding of off-label mt-sDNA testing. Future investigations into the inappropriate use of mt-sDNA will be crucial for curtailing the issue. Enhanced patient counseling, provider education, and healthcare initiatives are imperative for our ability to mitigate the human toll of CRC.
